# Spastic paraplegia-linked phospholipase *PAPLA1* is necessary for development, reproduction, and energy metabolism in *Drosophila*

**DOI:** 10.1038/srep46516

**Published:** 2017-04-19

**Authors:** Martina Gáliková, Peter Klepsatel, Judith Münch, Ronald P. Kühnlein

**Affiliations:** 1 Max Planck Institute for Biophysical Chemistry, Research Group Molecular Physiology, Am Faßberg 11, D-37077 Göttingen, Germany; 2 Max Planck Institute for Biophysical Chemistry, Department of Molecular Developmental Biology, Am Faßberg 11, D-37077 Göttingen, Germany; 3 Stockholm University, Department of Zoology, Svante Arrhenius väg 18B, S-106 91 Stockholm, Sweden; 4 University of Leipzig, Faculty of Chemistry and Mineralogy, Johannisallee 29, D-04103 Leipzig, Germany; 5 University of Graz, Institute of Molecular Biosciences, Humboldtstraße 50/2.OG, A-8010 Graz, Austria

## Abstract

The human PAPLA1 phospholipase family is associated with hereditary spastic paraplegia (HSP), a neurodegenerative syndrome characterized by progressive spasticity and weakness of the lower limbs. Taking advantage of a new *Drosophila PAPLA1* mutant, we describe here novel functions of this phospholipase family in fly development, reproduction, and energy metabolism. Loss of *Drosophila* PAPLA1 reduces egg hatchability, pre-adult viability, developmental speed, and impairs reproductive functions of both males and females. In addition, our work describes novel metabolic roles of PAPLA1, manifested as decreased food intake, lower energy expenditure, and reduced ATP levels of the mutants. Moreover, PAPLA1 has an important role in the glycogen metabolism, being required for expression of several regulators of carbohydrate metabolism and for glycogen storage. In contrast, global loss of PAPLA1 does not affect fat reserves in adult flies. Interestingly, several of the PAPLA1 phenotypes in fly are reminiscent of symptoms described in some HSP patients, suggesting evolutionary conserved functions of PAPLA1 family in the affected processes. Altogether, this work reveals novel physiological functions of PAPLA1, which are likely evolutionary conserved from flies to humans.

Phospholipases constitute an important class of enzymes, which serves a wide range of functions from digestive lipolysis, to membrane remodeling and cell signaling. Consistent with their fundamental functions, impaired phospholipase activity in humans is associated with numerous diseases including diabetes, cancer, neurodegenerative and neuromuscular diseases, which identifies phospholipases as important therapeutic targets^1^. All phospholipases catalyze the cleavage of a fatty acid from the glycerol-3-phosphate backbone of phospholipids. Depending on the fatty acid cleavage position, phospholipases are further categorized into sub-groups, with phospholipases A1 (PLA1) cleaving preferentially at the *sn*-1 position to generate free fatty acid and a 2-acyl-lysophospholipid. Interestingly, many PLA1 class enzymes, including *Manduca sexta* TGL and *Drosophila melanogaster* PAPLA1^2^ have an extended substrate spectrum and next to phospholipids also hydrolyze neutral lipids such as di- and triacylglycerol (TAG)^1^. The PLA1 sub-family of phospholipases is evolutionary highly conserved from yeast to mammals^1^,^3^,^4^. Depending on their specific functions, PLA1 enzymes act either intra- or extracellularly. Whereas extracellular, secreted PLA1s typically function as digestive enzymes, intracellular PLA1s (iPLA1s) mediate membrane remodeling and trafficking, and produce lysophospholipids with signaling functions^1^,^3^,^4^. Genomes of invertebrates typically code for a single iPLA1, also called PAPLA1 (for Phosphatidic Acid PLA1), while mammalian iPLA1/PAPLA1 family consists of three members: DDHD1 (or PAPLA1), DDHD2 (or KIAA0725p), and the SEC23 interacting protein (SEC23IP or p125)^3^,^4^.

Mutations in DDHD1^5^ and DDHD2^6^ are in humans associated with hereditary spastic paraplegia (HSP), a genetically heterogeneous syndrome with progressive spasticity and weakness of lower limbs as the lead symptom^7^,^8^. The disease mechanism underlying the malfunction of the limbs is supposed to be the progressive degeneration of the pyramidal tract axon^7^,^9^. Onset of this degeneration varies; early onset is often associated with mild progression without the need for wheelchair assistance. Later disease onset leads frequently to loss of walking ability^10^,^11^. While DDHD1 mutations result in the pure, non-complicated form of HSP (type SPG28)^12^, DDHD2 is associated with the complex form of the disease (type SPG54)^13^. The SPG28 type of HSP is thus clinically characterized by weakness of the lower limbs and urinary sphincter dysfunction only, whereas the SPG54 type can be accompanied by a variety of additional neurological defects such as ataxia, mental retardation, fecal incontinence and others^8^,^9^. Apart from the neurological and locomotor symptoms, knowledge of other physiological and metabolic changes associated with HSP is rather limited. For example, the most frequent form of HSP, which is caused by mutations in the *SPG11* gene, is associated with metabolic disorders such as dysphagia and mobility-independent obesity of unknown etiology^14^. Interestingly, dysphagia and developmental delay were also observed in some of the patients with HSP linked to DDHD2 deficiency^6^,^15^, suggesting that PAPLA1 family might have currently uncharacterized developmental and metabolic roles. However, understanding of the developmental and metabolic consequences of iPLAs deficiencies in human patients is hampered by various limitations including the low frequency of the disease, the variable penetrance and expressivity of the associated symptoms, and the confounding effects of the altered lifestyle of the patient. Yet, knowledge of the physiological effects of the iPLA1 deficiencies is important also for the disease management.

Thus, *PAPLA1* mutant animal models promise valuable insights into additional roles of iPLAs in health and disease. Surprisingly, DDHD1^16^ and SEC23IP^17^ mutant mice models show only minor physiological defect, i.e. male subfertility^16^,^17^. More severe phenotypes might be masked by functional redundancy among the mammalian PAPLA1 family members. Accordingly, less complex model systems that encode a single *PAPLA1* gene might prove informative for a comprehensive understanding of the cellular, physiological and developmental functions of this enzyme family.

*Drosophila melanogaster* is a very powerful model system, which has been successfully used to study a variety of muscular, neuromuscular and neurodegenerative disorders, including HSP caused by mutations in *spastin*^18^,^19^,^20^ or KIF5A^21^. With respect to *PAPLA1*, three elegant studies proved the value of the fly model to study iPLA1- linked HSPs. Firstly, Liebl *et al*. showed neuromuscular junctions overgrowth caused by transposon insertion at the *PAPLA1* gene region^22^. Later work by Schuurs-Hoeijmakers and colleagues demonstrated that RNAi-mediated knock down of *PAPLA1* decreases active zones of neuromuscular junction, which causes synaptic transmission impairment similar to what occurs in the HSP patients^6^. Recently, Kunduri *et al*. proved that loss of fly *PAPLA1* results in age-dependent progressive impairment of climbing ability^23^, reminiscent of progressive walking impairment accompanying HSP in humans^7^. Interestingly, this study indicated that, next to its role in the synaptic transmission, *PAPLA1* might have much broader roles in fly physiology. The protein is involved in COPII transport, posttranslational modifications, and vesicular transport of G protein-coupled receptors (GPCRs). Given the plethora of signals that are transduced by GPCRs^24^,^25^, loss of *PAPLA1* can be expected to severely affect fly physiology, including developmental and metabolic roles. In addition, an *in vitro* study of fly *PAPLA1* activity has shown that this enzyme has also TAG lipase activity; and its homolog in *Manduca sexta* has been portrayed as the main TAG lipase in insects in general^2^. However, neither lipolytic, nor developmental nor metabolic roles of *PAPLA1* have been addressed *in vivo* so far.

We thus reasoned that implementation of *Drosophila* as a model for human diseases caused by defective iPLA1s requires a general understanding of the physiological and metabolic consequences of *PAPLA1* deficiency.

Our work indeed reveals broad physiological roles of *PAPLA1* in embryonic and larval development, developmental timing, reproduction of both males and females, and in general energy metabolism. We show that impairment of energy-demanding processes such as reproduction and locomotion of *PAPLA1* mutants is accompanied by reduction of food intake. Low energy intake is nevertheless antagonized by reduced basal metabolic rate. However, ATP reserves of *PAPLA1* mutants remain considerably low. Interestingly, our study shows that despite the prediction of *PAPLA1* coding for the main insect lipase^2^, deficiency for this enzyme does not increase fat storage. Nevertheless, *PAPLA1* has important roles in the carbohydrate metabolism. Altogether, using *Drosophila* as a model system, we describe the broad developmental and metabolism-related role of this enzyme, which might be present, but due to the gene redundancy partially masked also in the mammalian family of PAPLA1 enzymes.

## Results

### Generation of *PAPLA1*-deficient flies by CRISPR/Cas9-mediated genome engineering

**Figure 1:**
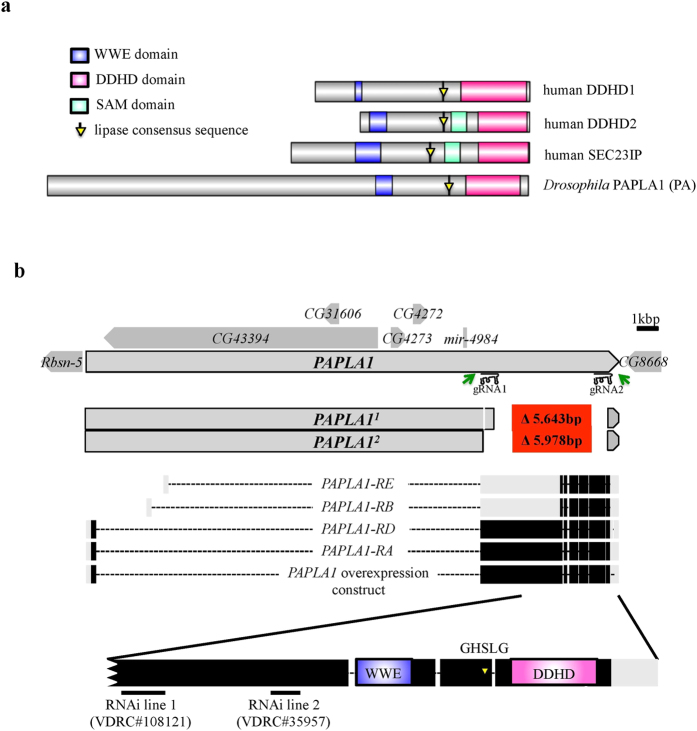
Structure and functional domains of the human and *Drosophila* PAPLA1 members, molecular identity of the fly *PAPLA1* mutants, and schematic representation of other genetic tools used to study the gene functions. **(a)** Human and fly PAPLA1 members and their functional domains. **(b)** The *Drosophila PAPLA1* locus at chr. 2 L is flanked by the genes *Rbsn-5* and *CG8668* and encompasses additional five genes (*CG43394, CG31606, CG4272, CG4273* and *mir-4984*; dark grey). FlyBase (FlyBase release r6.06) annotates four *PAPLA1* transcript isoforms (RA to RD; light grey = non-coding exons; black = coding exons) *PAPLA1*^*1*^ and *PAPLA1*^*2*^ deletion mutants were induced by CRISPR/Cas9-assisted genome editing with gRNAs 1 and 2 flanking all main PAPLA1 domains shared by all protein isoforms. The *PAPLA1*^*1*^ allele has a short 6 nt deletion (pos. 2 L:8140678–8140673), and a large 5.643 bp deletion (pos. 2 L: 8140324–813468), with two additional nucleotides (AT) in-between the breakpoints. *PAPLA1*^*2*^ represents a 5.978 kbp deletion (pos. 2 L: 8140659–8134682), with a short ectopic sequence (CCATCCA) between the breakpoints (REFSEQ:NT_033779; FlyBase release r6.06). Sequence of the other genes encoded by the *PAPLA1* locus remains intact in both mutant alleles. Green arrows indicate the binding sites of primers that were used for genotyping. To study the *PAPLA1* gain-of function, we created UAS-*PAPLA1* overexpression line, allowing overexpression of the full-length *PAPLA1* identical to the RA transcript. Black bars depict the targeting positions of the two RNAi lines used in this study.

To analyze the biological roles of *PAPLA1* in the *Drosophila* model system, we first created mutants deficient for all of the functional domains that are conserved in the mammalian iPLA1s, i.e. the DDHD and WWE domain, and the GHSLG sequence, which matches the canonical lipase consensus sequence GxSxG (Fig. 1a). Since the genomic region of fly *PAPLA1* is exceptionally large and codes for five additional genes (Fig. 1b), we specifically targeted the genome region that is unique to *PAPLA1*. The corresponding deletion was triggered by simultaneous mutagenesis of exon 2 and exon 7 (Fig. 1b) by co-expression of two gRNAs and a source of CAS9 in the male germline according to Kondo and Ueda^26^. This approach led to two independent deletion alleles, which were termed *PAPLA1*^*1*^ and *PAPLA1*^*2*^ (Fig. 1b). Both deletions represent functional null alleles. These deletion mutants were backcrossed into a common genetic background for nine generations prior to the analysis (for details see Materials and Methods).

### *PAPLA1* is necessary for pre-adult development

**Figure 2:**
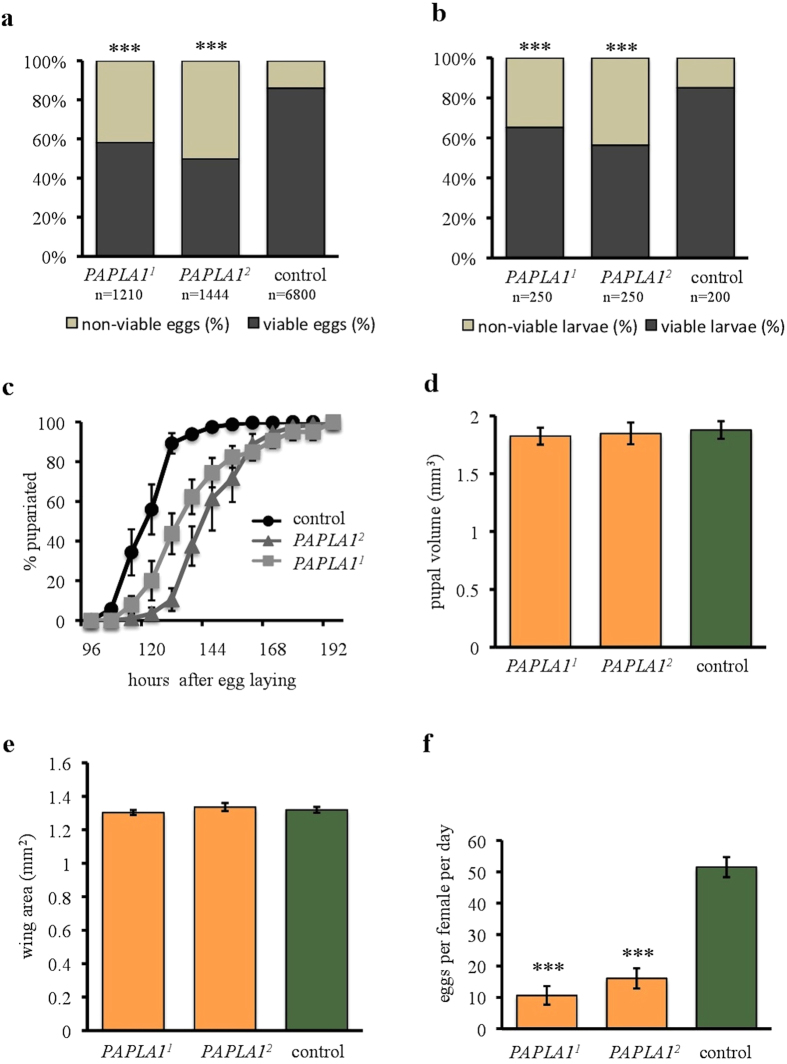
Developmental and reproduction-related roles of *PAPLA1*. (**a**) *PAPLA1* deficiency reduces hatchability (egg to 1^st^ larval instar survival). Fischer’s exact test: *P* < 0.001. **(b)** *PAPLA1* deficiency reduces viability (survival from the 1^st^ larval instar to adulthood). Larvae that did not give rise to adult flies are denoted as non-viable. Fischer’s exact test: *P* < 0.001. **(c)** *PAPLA1* deficiency reduces developmental speed. Shown are mean % of pupariated flies at the given time point ± SEM. Mann Whitney test to compare median developmental times: *P* < 0.001 for both *PAPLA1* mutants. **(d)** *PAPLA1* deficient flies develop into pupae with standard volume. Two-tailed Student’s *t*–test: *P* > 0.05. **(e**) *PAPLA1* deficiency does not affect body size of adults (measured as wing area). Two-tailed Student’s *t*–test: *P* > 0.05 for both assays. **(f)** *PAPLA1* deficient flies have lowered egg production. Two-tailed Student’s *t*–test: *P* < 0.001.

Firstly, we tested the potential developmental roles of *PAPLA1. PAPLA1*^*1*^ and *PAPLA1*^*2*^ mutations resulted in reduced hatchability, measured as proportion of eggs that developed into the 1^st^ instar larvae (Fig. 2a). Viability of the mutants, estimated as the proportion of 1^st^ instar larvae that successfully developed into adulthood, was reduced, too (Fig. 2b). Moreover, developmental speed of *PAPLA1* mutants, estimated as the time from the egg deposition until the puparium formation, was considerably reduced (Fig. 2c). Altogether, ontogenetic defects of the tested mutants imply that the PAPLA1 family has important developmental functions.

### *PAPLA1* has reproduction-associated functions in males and females

**Figure 3:**
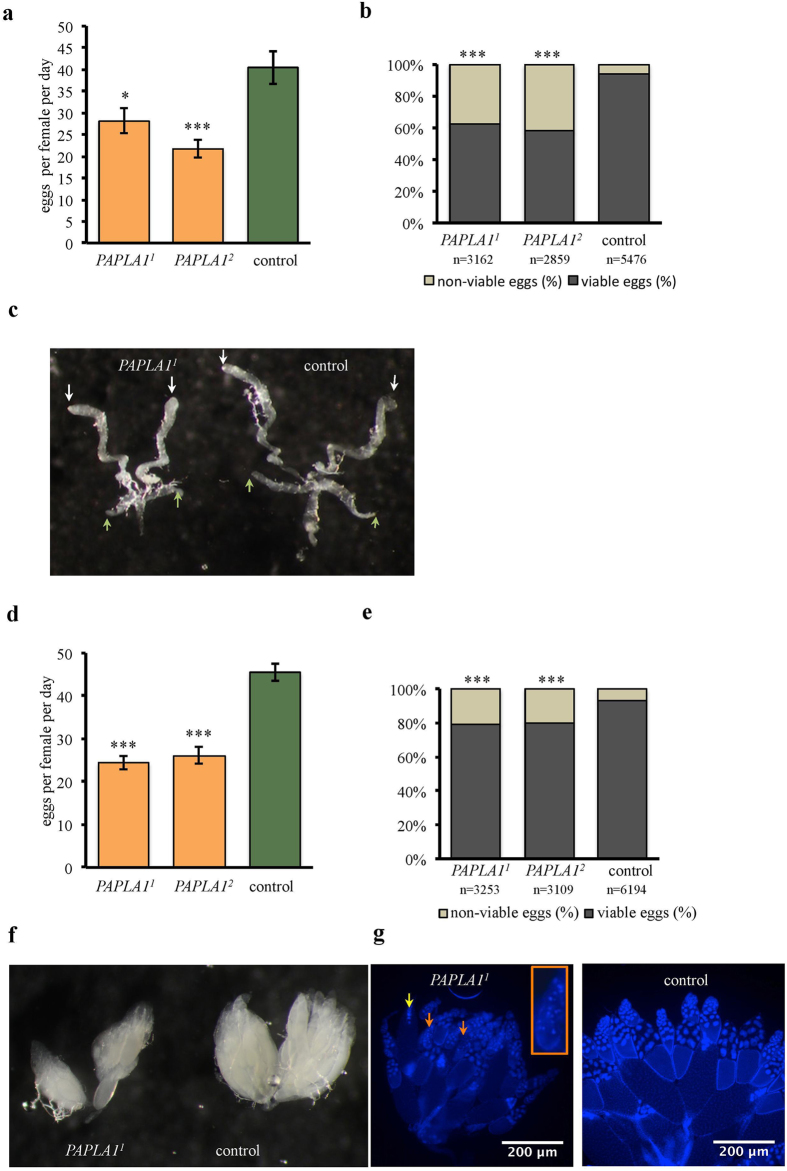
*PAPLA1* has important reproduction-related roles in both males and females. **(a)** *PAPLA1* function in males is necessary to stimulate normal egg laying in tester females. Two-tailed Student’s *t*–test: *PAPLA1*^*1*^ vs. control *P* < 0.05, *PAPLA1*^*2*^ vs. control *P* < 0.001. **(b)** *PAPLA1* deficient males are subfertile. Sub-fertility of *PAPLA1* deficient males shown by low hatchability of eggs produced upon mating with tester females. Tested is the ratio between the viable and non-viable eggs produced by single pair crosses upon mating with tester females. Fischer’s exact test: *P* < 0.001. (**c**) Hypotrophy of male reproductive organs in *PAPLA1* mutants; note the smaller size of testes (white arrows) and of accessory glands (yellow arrows). (**d**–**g**) *PAPLA1* is required for female reproduction. (**d**) *PAPLA1* mutant females have reduced fecundity when mated with tester males. Two-tailed Student’s *t*–test: *P* < 0.001. **(e)** *PAPLA1* deficiency in females decreases egg hatchability. Fischer’s exact test: *P* < 0.001. **(f)** *PAPLA1* deficient females have smaller ovaries with reduced number of vitellogenic stage 10 chambers (see also **g**). **(g**) Note the occasional degeneration of egg chambers at the mid-oogenesis checkpoint (orange arrows, detail of the degenerating chamber in orange-lined rectangular). Stage 14 egg chambers occasionally contain nurse cell nuclei (yellow arrow).

Despite the extended developmental time, *PAPLA1*^*1*^ and *PAPLA1*^*2*^ mutant flies reached normal size, documented by standard volume of pupae (Fig. 2d) and wing area of adults (Fig. 2e). Interestingly, egg production of homozygous *PAPLA1-*deficient parents was dramatically reduced (Fig. 2f), indicating important reproduction-related functions of this gene. To differentiate between the male- and female-specific requirements of *PAPLA1* in reproduction, we first mated *PAPLA1*-deficient males with virgin *PAPLA1*^+^ tester females. These tester females laid considerably less eggs (Fig. 3a) than tester females mated with control males, indicating that the gene has a male-specific role in inducing female ovulation. In line with the previously described requirement of *PAPLA1* for spermatogenesis^23^, hatchability of eggs laid by the tester females mated to *PAPLA1* mutants was significantly decreased, too (Fig. 3b). Males induce ovulation in females by proteins that are produced in the male accessory glands, and deposited into females during copulation^27^. Consistent with the reduced capacity to induce ovulation when mated to tester females, *PAPLA1*^*1*^ and *PAPLA1*^*2*^ mutant males had smaller accessory glands (Fig. 3c). In addition, testes of the *PAPLA1* deficient males were smaller as well (Fig. 3c), as reported previously^23^, suggesting a general role of *PAPLA1* in the development of male reproductive tract.

To test the potential role of *PAPLA1* in female reproduction, we crossed virgin *PAPLA1* deficient females with *PAPLA1*^*+*^ tester males. Oviposition of the mutant females was considerably reduced (Fig. 3d). Moreover, hatchability of eggs laid by the *PAPLA1*^*1*^ and *PAPLA1*^*2*^ mutant females was mildly but significantly reduced (Fig. 3e), suggesting that *PAPLA1* regulates egg production also qualitatively. Ovaries of *PAPLA1* females appeared normal without any severe malformations, just smaller due to the reduced number of vitellogenic egg chambers (Fig. 3f,g), suggesting reduced speed of egg production. We also noted increased degeneration of egg chambers at the mid-oogenesis stage (Fig. 3g, orange arrows, for detail see also the enlarged degenerating chamber in the orange rectangle), which is a hallmark of nutritional stress^28^, and occasional persistence of nurse cell nuclei after stage 13 of oogenesis (Fig. 3g, yellow arrow).

Altogether, our work revealed novel roles of *PAPLA1* in *Drosophila* reproduction; in addition to its known role in spermatogenesis^23^, male *PAPLA1* is required also for proper development of accessory glands, and for male ability to induce female ovulation upon mating. In female reproduction, *PAPLA1* is required for both oogenesis rate and viability of the produced egg.

### *PAPLA1* controls general energy homeostasis

**Figure 4:**
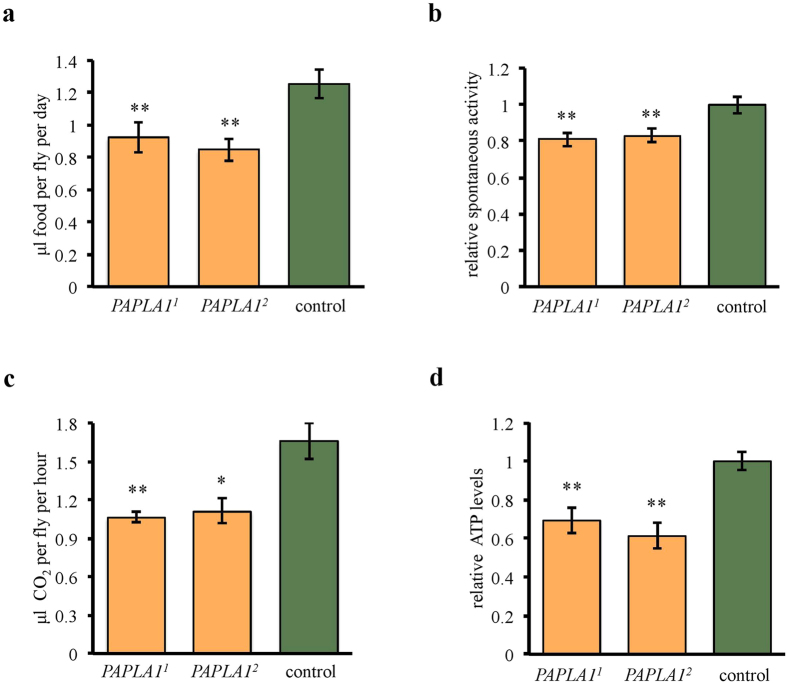
*PAPLA1* deficiency reduces both energy intake and energy expenditure. (**a**) *PAPLA1*^*1*^ and *PAPLA1*^*2*^ mutants have reduced food intake. (**b**) Spontaneous locomotor activity of *PAPLA1*^*1*^ and *PAPLA1*^*2*^ mutants is reduced. (**c**) Metabolic rate (estimated as CO_2_ production) of *PAPLA1*^*1*^ and *PAPLA1*^*2*^ mutants is reduced. **(d**) ATP levels of *PAPLA1*^*1*^ and *PAPLA1*^*2*^ mutants are reduced. All panels: Two-tailed Student’s *t*–test: *P < 0.05; **P < 0.01.

Since reproduction is an energetically demanding process, we hypothesized that the reduced reproductive success of *PAPLA1* mutants might be at least partially caused by a general impairment of energy metabolism. Moreover, some patients with the DDHD2–associated spastic paraplegia have dysregulated food intake^6^,^14^, also suggesting defects of energy balance in the absence of PAPLA1 enzymes. Thus, we aimed to test whether *PAPLA1* deficiency affects energy intake in flies. Monitoring the food intake by capillary feeding assay revealed significant reduction in daily food intake in both *PAPLA1*^*1*^ and *PAPLA1*^*2*^ mutants (Fig. 4a). Next, we measured activity-dependent energy expenditure, indicated by spontaneous locomotion. Spontaneous walking, monitored over a week period, was reduced in the mutants, too (Fig. 4b). Consistently, the CO_2_ production, which is a measure of the metabolic rate, was also decreased upon *PAPLA1* loss-of-function (Fig. 4c). In addition, the lower metabolic rate was accompanied by considerably decreased level of ATP (Fig. 4d).

Altogether, our data show that *PAPLA1* deficiency reduces both food intake and energy expenditure, decreasing energy turnover in general. Reduced metabolic capacity of the *PAPLA1* mutants thus likely contributes to the observed impairment of energetically demanding processes such as reproduction and locomotion.

### Differential effects of *PAPLA1* on body fat storage

**Figure 5:**
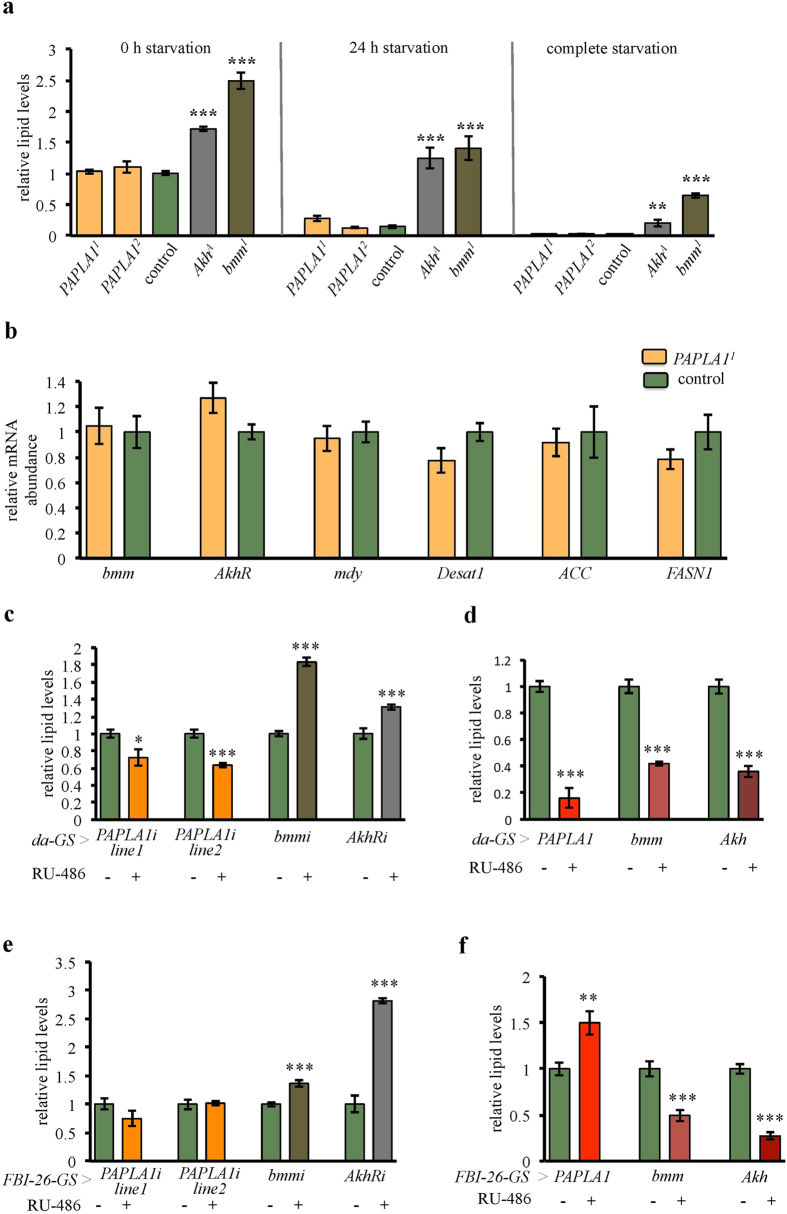
*PAPLA1* deficiency does not increase fat storage nor affects lipid mobilization. **(a)** *PAPLA1* mutants store normal fat reserves and are able to mobilize them completely. Note the difference to the *Akh*^*A*^ and *bmm*^*1*^ mutants. Lipid content was determined prior to starvation, after 24 h of starvation, and after complete starvation. Lipid levels were normalized to the protein levels at the given time point, and standardized to the relative lipid levels of control flies prior to starvation. (**b**) Consistent with normal body fat storage, expression of lipolysis (*bmm, AkhR*) and lipogenesis (*mdy, Desat1, ACC, FASN1*) genes in *PAPLA1* mutants is normal. (**c**) Adulthood-specific ubiquitous knockdown of *PAPLA1* driven by *daughterless*-GeneSwitch (*da*-GS) decreases fat storage. Note the contrast to the corresponding knockdown of lipolytic genes *bmm* and *AkhR*. (**d**) Adulthood-specific overexpression of wild type *PAPLA1* reduces lipid levels, similarly as overexpression of lipolytic genes *bmm* and *AkhR*. (**e**) Adulthood-specific knockdown of *PAPLA1* in the fat body driven by the *FBI-26*-GeneSwitch (*FBI-26*-GS) driver does not affect fat storage. Two independent RNAi lines were used. Note the contrast to the obesogenic effects of the corresponding knockdown of the lipolytic genes *bmm* and *AkhR*. (**f**) Adulthood-specific overexpression of *PAPLA1* in the fat body considerably increases fat storage. Note the contrast to the lean phenotype triggered by overexpression of *Akh* or *bmm*. All panels: Two-tailed Student’s *t*–test: *P < 0.05; **P < 0.01; ***P < 0.001.

The above-described defects in the energy metabolism, and the prediction that *PAPLA1* encodes the main insect TAG lipase^2^ prompted us to address a potential role of the gene in the energy reserves. In order to test the potential roles of *PAPLA1* in the fat storage, we compared the *PAPLA1* mutants to the lipolysis-defective *bmm*^*1*^ 29 and *Akh*^*A*^ 30 mutants. These mutants are obese and display incomplete storage lipids mobilization under starvation^29^,^30^. To exclude genetic background effects on the fat storage, these mutants were backcrossed into the same genetic background as the *PAPLA1* mutants. In contrast to *bmm*^*1*^ and *Akh*^*A*^ flies, fat storage in *PAPLA1*^*1*^ and *PAPLA1*^*2*^ mutants was normal, and they completely mobilized their lipid reserves upon starvation (Fig. 5a). To exclude that a potential role of *PAPLA1* in body fat storage is masked by compensatory changes in *PAPLA1* mutants, we tested the expression levels of several key regulators of fat storage that are known to be transcriptionally regulated. Consistent with their normal fat storage, *PAPLA1* mutants had normal mRNA levels of the key lipid catabolism genes *bmm* and *Akh* receptor (*AkhR*) (Fig. 5b). Similarly, expression levels of the lipogenesis genes *midway (mdy*; encoding *Drosophila* diacylglycerol acyltransferase 1), *Desaturase 1 (Desat1*), *Acetyl-CoA carboxylase (ACC*), and *Fatty acid synthase 1 (FASN1*) were unaffected in *PAPLA1* loss-of-function mutants (Fig. 5b).

Taken together, *PAPLA1* mutants are able to adapt to the lower ATP levels and energy flux without compromising their fat storage. Next we investigated fat storage in flies with partial knock-down of *PAPLA1*. We used two independent RNAi lines, both of which significantly reduced *PAPLA1* mRNA levels (Supplementary Fig. S1), and induced their expression in adult flies using the ubiquitous *daughterless*-GeneSwitch (*da*-GS) driver^31^. Adult-specific *PAPLA1* knockdown reduced fat storage, opposite to the obese phenotype caused by the corresponding manipulation of the lipid catabolism genes *AkhR* and *bmm*^32^ (Fig. 5c). Nevertheless, *da-*GS-driven overexpression of wild type form of *PAPLA1* decreased fat content (Fig. 5d), similar to overexpression of *Akh*^32^ or *bmm*^29^. Yet the low fat content triggered by this manipulation might have been contributed also by the toxicity of the manipulation, as around 50% of flies died prior to the analysis. Next, we tested whether the observed leanness in response to the ubiquitous *PAPLA1* RNAi is due to an organ-specific function of the gene in the fat body or rather a secondary consequence of functional impairment of *PAPLA1* in non-adipose tissues. Therefore, we knocked down PAPLA1 exclusively in the fat body, using the *FBI-26-GeneSwitch* driver (*FBI-26*-GS)^33^. Fat body-specific RNAi targeting of *AkhR* or *bmm* phenocopies the obesity of the respective null mutants (Fig. 5e). In contrast, impairment of *PAPLA1* function in the fat body did not affect the body fat content neither in response to gene knockdown during adulthood (*FBI-26*-GS; Fig. 5e), nor when driven throughout the development (*Lpp*-GAL4; Supplementary Fig. S2). Interestingly, *PAPLA1* overexpression in the fat body even increased the fat reserves, contrasting with the lean phenotype caused by overexpression of *AkhR* or *bmm* (Fig. 5f).

Taken together, *PAPLA1* deletion mutants exhibit normal fat storage and mobilization. Yet, conditional gene knockdown experiments revealed a role of *PAPLA1* in the fat storage control, which might be compensated in the null mutant situation. Importantly, none of the tested modulations of *PAPLA1* mimiced the fat storage changes caused by the corresponding manipulations of central lipid mobilization genes.

### *PAPLA1* regulates glycogen storage

**Figure 6:**
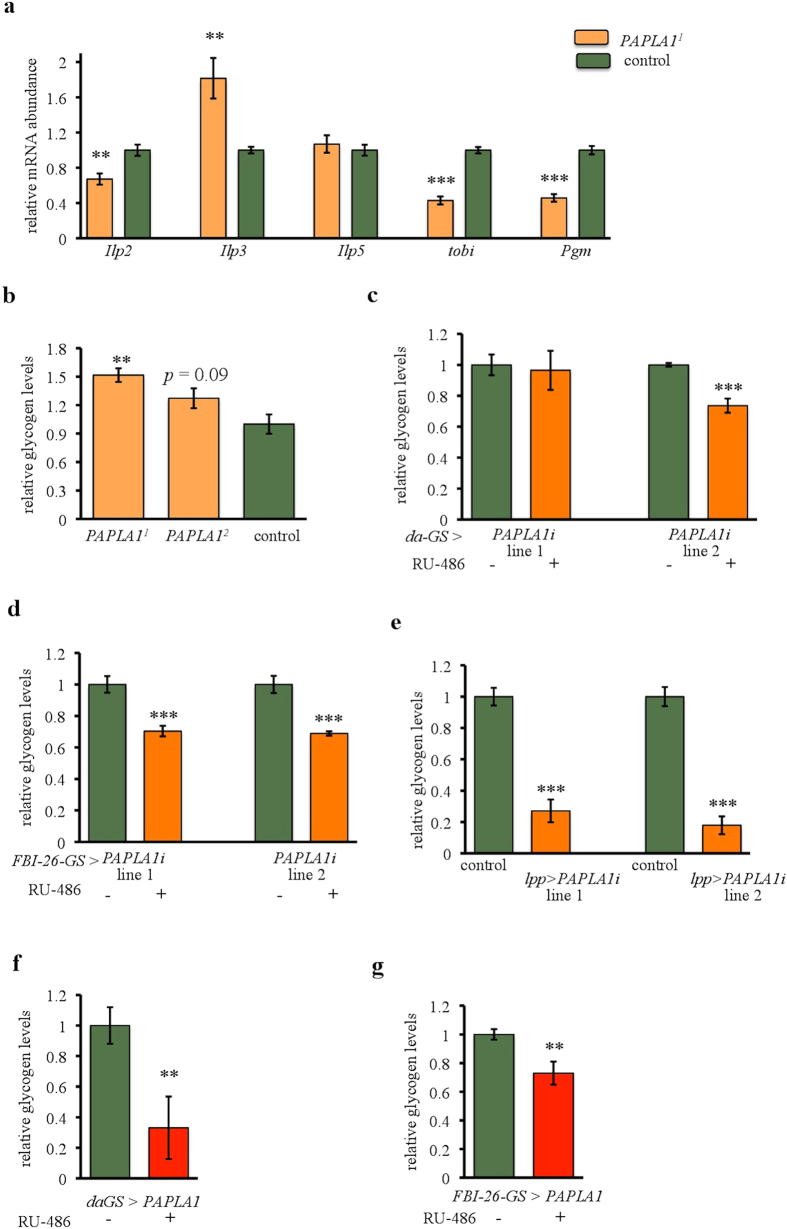
*PAPLA1* regulates glycogen storage and controls the expression of carbohydrate metabolism regulators. **(a)** *PAPLA1* mutants have mis-regulated expression of genes involved in the carbohydrate metabolism. Expression of *Ilp2* is reduced, whereas expression of *Ilp3* increased. Expression of genes encoding enzymes involved in glycogen cleavage, *tobi* and *Pgm*, is reduced. **(b)** *PAPLA1* mutants have increased glycogen storage. **(c)** Ubiquitous, adulthood-specific knockdown of *PAPLA1* by *daughterless*-GeneSwitch (*da*-GS) decreases glycogen storage when the stronger RNAi line is used (i.e. line 2; see also Supplementary Fig. S1). The weaker RNAi line (line 1) has no significant effect on glycogen storage. **(d)** Fat body targeted, adulthood-specific *PAPLA1* knockdown by *FBI-26-*GeneSwitch (*FBI-26-GS*) reduces glycogen content. **(e)** Fat body targeted *PAPLA1* RNAi driven by *Lpp-*Gal4 dramatically reduces glycogen content. **(f)** Adulthood-specific overexpression of *PAPLA1* driven ubiquitously from the *daughterless*-GeneSwitch (*da*-GS) decreases glycogen storage. **(g)** Adulthood-specific overexpression of *PAPLA1* in the fat body, driven by the *FBI-26*-GeneSwitch (*FBI-26*-GS), decreases glycogen storage. All panels: Two-tailed Student’s *t*–test: **P < 0.01; ***P < 0.001.

While fat is the calorically most important energy reserve, carbohydrates stored in the form of glycogen constitute the second pillar of energy storage in flies. Carbohydrate metabolism is regulated primarily by the insulin/insulin-like pathway^34^,^35^. Hence, we firstly tested the expression of fly Insulin-like peptides (Ilps). Expression of *Ilp2* was reduced and *Ilp3* was up-regulated, while *Ilp5* remained unchanged in the *PAPLA1* mutants (Fig. 6a). However, downstream targets of insulin-like signaling, such as *Thor* and *l*(*2*)*efl*, were not changed (Supplementary Fig. S3). Nevertheless, consistent with low levels of *Ilp3*, expression of *target of brain insulin (tobi*) was reduced in *PAPLA1* mutants (Fig. 6a). The *tobi* gene codes for an evolutionary conserved α-glucosidase, which is positively regulated by insulin signaling^36^. This enzyme is involved in glycogen catabolism, cleaving the polysaccharide and releasing free glucose molecules. Similarly to *tobi*, expression of *phosphoglucose mutase (Pgm*), another enzyme acting downstream of glycogen cleavage, was also reduced in the absence of *PAPLA1* (Fig. 6a). This suggests lowered glycogen catabolism in *PAPLA1* mutants. Consistently, glycogen levels of *PAPLA1* mutants were indeed increased (Fig. 6b).

Considering the developmental and tissue-specific effects of PAPLA1 function on fat storage (Fig. 5), we next tested the effect of conditional *PAPLA1* gene knockdown on glycogen reserves. Opposite to the *PAPLA1* null phenotype, adulthood-specific ubiquitous *PAPLA1* RNAi decreased glycogen reserves (Fig. 6c), and so did the fat body-specific knock down, irrespective whether conducted during adulthood only (Fig. 6d) or throughout the whole ontogenesis (Fig. 6e). Similar to RNAi, overexpression of the wild type isoform of *PAPLA1* also decreased glycogen content when driven ubiquitously (Fig. 6f) or specifically in the fat body (Fig. 6g). Thus, both low and high levels of *PAPLA1* in the fat body affect glycogen storage.

Collectively, our experiments provide the first evidence that an enzyme from the *PAPLA1* family is required for proper carbohydrate metabolism. The impact of PAPLA1 function on carbohydrate but also on fat storage depends on the timing/strength of manipulation of the gene, suggesting existence of compensating mechanisms that underlie the energy homeostasis function of *PAPLA1* in the fly.

## Discussion

Given their roles in fundamental cellular processes such as membrane dynamics and trafficking, PAPLA1 enzymes are expected to serve a wide spectrum of physiological functions^1^,^3^,^4^. However, analysis of these roles in mammals might be complicated by redundancy among the multiple mammalian enzymes. Consistently, mice models lacking a single PAPLA1 family gene result in a surprisingly subtle phenotype^4^,^16^,^17^. This study aimed to investigate *PAPLA1* functions using the *Drosophila* model, which has only a single member of this family, and is thus free of any confounding effects of redundancy that might occur in the mammals. Taking advantage of this model system we show that PAPLA1 is required for a broad range of functions, from early development to energy storage. Interestingly, *PAPLA1* deficiency is not completely lethal in flies, suggesting that other mechanisms might partially compensate for its function. *PAPLA1* mutants develop into normally sized adults, nevertheless, they have severely reduced metabolism, with lowered food intake and energy expenditure. This metabolic impairment likely contributes to the decreased spontaneous movement and to the lower reproductive success observed in these mutants. Subfertility of the *PAPLA1*^*1*^ deficient flies correlates well with the spermatogenesis defects of mutants in its mammalian homologs Sec23IP^4^,^17^ and Ddhd1^16^. This phenotype can be partially attributed to the previously reported spermatid individualization defects in flies lacking *PAPLA1* function^23^, but also to the here-reported hypotrophy of accessory glands. Remarkably, the small size of both the testes and the accessory glands in *PAPLA1* mutant flies is reminiscent of the hypogonadism that was described for the spastic paraplegia caused by deficiency in the phospholipase PNPLA6^37^. In *Drosophila*, male fertility depends on spermatogenesis, but also on products of the accessory glands called sex peptides, which are transferred to females during copulation^27^. These peptides are necessary to induce ovulation and behavioral changes in females upon mating, maximizing thus the reproductive success of the male^27^. We speculate that loss of *PAPLA1* interferes with sex-peptide function, as *PAPLA1*^*1*^ and *PAPLA1*^*2*^ males fail to stimulate oviposition when mated to females with normal PAPLA1 function. Thus, *PAPLA1* deficiency may reduce male reproductive success in two ways – by impairing the production of sperm and by failure to induce standard ovulation in the mated females. Notably, whereas Kunduri *et al*. reported complete sterility of their *PAPLA1* knockout males^23^, we observed severe subfertility, but not complete loss of the reproductive capacity of *PAPLA1*^*1*^ and *PAPLA1*^*2*^ mutant males. This suggests that the penetrance of the phenotype is likely modulated by genetic background and/or on environmental conditions. Intriguingly, these factors might also be responsible for the variability of symptoms in human patients suffering from *PAPLA1* family-linked HSP.

Next to the known role in male reproduction, our work establishes for the first time the role of a *PAPLA1* member in female reproduction. Interestingly, the decrease in oogenesis with occasional degeneration of egg chambers at the nutritionally controlled mid-oogenesis checkpoint is reminiscent of the same phenotypes triggered in wild type flies by nutritional shortage^28^. Since the *PAPLA1* mutation reduces general metabolism and ATP levels, these metabolic defects likely also contribute to the reduced reproduction of the mutants.

Deficiency for *PAPLA1* causes a complex metabolic phenotype suggestive of low metabolic flux; hypophagia, low basal metabolism and reduced spontaneous locomotion. The primary cause of this metabolic reduction is not known; however, several lines of evidence suggest that these defects might be triggered by impaired mitochondrial function. Recent work showed that Yor022c, the yeast homolog of *PAPLA1*, regulates mitochondrial dynamics and is thus required for the proper ATP production^38^. Moreover, impaired spermatogenesis of *PAPLA1*-deficient mice is also associated with reduced mitochondrial function^16^. Finally, the DDHD1-linked HSP in some human patients is also associated with lowered mitochondrial function^39^, decreased oxidative metabolism, and reduced ATP levels^5^. Our corresponding findings in mutant flies suggest, that PAPLA1 enzymes have a role in oxidative metabolism and ATP production that is evolutionarily conserved from yeast to human. Moreover, mitochondrial dysfunction and low basal metabolic rate might be serious, if not the principal cause of the reduced reproductive capacity and spontaneous movement of *PAPLA1* mutants.

Interestingly, fat stores in *PAPLA1* mutant flies are normal, suggesting that the reduced metabolic rate antagonizes the low food intake. On the other hand, normal fat storage of the fly mutants is surprising in view of an *in vitro* study showing that *PAPLA1* encodes the main fat body TAG lipase of the tobacco hornworm *Manduca sexta*, and that a truncated *Drosophila melanogaster* PAPLA1 has TAG lipase activity as well^2^. Our work emphasizes the importance of genetic manipulations in investigations of the physiological roles of a gene, as neither *PAPLA1* deficiency nor conditional *PAPLA1* gene knockdown increases lipid stores in flies, whereas adipose tissue-specific *PAPLA1* over-expression results in obesity. However, while *PAPLA1* deficiency does not affect global fat storage, we cannot exclude that the TAG lipase activity of PAPLA1 is required in certain tissues that do not contribute significantly to the total body fat storage. Such a role has been described for DDHD2 in the nervous system of mice^40^. Absence of *PAPLA1*-associated lipid storage phenotype is also surprising in view of PAPLA1 acting in the COPII-mediated vesicular transport^23^. Components of the COPII machinery have been previously identified as regulators of *Drosophila* fat storage in genetic screens^41^,^42^. Moreover, *PAPLA1* is required for post-translational processing and transport of GPCRs^23^, which mediate metabolic signaling via a broad spectrum of ligands including peptide hormones. As insect neuropeptides regulate numerous processes ranging from feeding to energy mobilization^25^, impaired GPCR signaling might underlie the hypophagia and reduced metabolic rate of the *PAPLA1* mutants.

To the best of our knowledge, our work provides the first evidence that an iPLA1 family enzyme is involved in glycogen storage. Interestingly, the effect of *PAPLA1* on carbohydrate storage depends on the range of the manipulation. Complete gene knockout in the null mutants increases glycogen storage, whereas ubiquitous or fat body-specific knockdown by RNAi in adult flies only has the opposite effect. We assume that *PAPLA1* deficiency activates compensatory mechanisms that counteract the mutation, but are not activated by partial, RNAi-mediated gene knockdown. Mechanism of genetic compensation, which ameliorates the effect of complete null, but not knockdown by RNAi, was described in zebrafish^43^ and recently reported^44^ and discussed^45^ also for *Drosophila*. However, it is also possible that the lack of stronger phenotypes in the *PAPLA1* mutants might simply result from the very general and broad role of *PAPLA1* in GPCRs transport. Given the plethora of ligands with metabolic functions that signal via GPCRs, it is possible that *PAPLA1* deficiency simultaneously affects antagonistically acting pathways, and the effect of impairment thus averages out. Nevertheless, temporally and/or spatially restricted *PAPLA1* gene knockdown might affect only part of the signaling network, shifting thus the signaling balance, and disclosing the role of the gene in the energy homeostasis.

Altogether, this study reveals novel, unexpected functions of PAPLA1, including roles in development and developmental timing, female reproduction, as well as metabolic functions such as regulation of food intake, metabolic rate, ATP production, and carbohydrate storage. These functions might be evolutionary conserved, but difficult to disentangle in mammalian models with potential functional redundancy among the PAPLA1 enzymes. Importantly, similar defects as we observed in the *PAPLA1* mutants might contribute to the complications accompanying HSP in humans. Of particular interest are the developmental delay and the dysphagia, which were also observed in the DDHD2-linked HSPs^6^,^15^. This suggests that requirement for *PAPLA1* in developmental progression and food intake is evolutionary conserved from flies to humans. Whether the additional here-described roles of *PAPLA1* in development, reproduction and metabolism are conserved in humans awaits clinical correlation studies. For many years, genetic causes of HSPs were studied only by linkage analysis. Yet times are changing thanks to the almost routine use of next-generation sequencing in clinical studies, which led to numerous reports of PAPLA1-family linked HSPs over the last years^13^,^46^,^47^, and to the discovery of PAPLA1-failure being involved also in some of the previously-reported linkage studies^6^. Nevertheless, even though clinical studies identify the disease-associated genes, their functional analysis is still dependent on the basic research in the model organisms. The case of the *PAPLA1* family-associated HSPs demonstrates that *Drosophila* can be even more informative than mammalian animal models, as only the fly mutant recapitulates some of the HSP-linked phenotypes like walking defects and impairment of the neuromuscular junctions^6^,^16^,^23^.

## Material and Methods

### Fly stocks and fly husbandry

Flies were reared at 25 °C in 12 h light – 12 h dark cycle under controlled density conditions. Details on the rearing conditions and diets are available in the Supplementary Information. List of the fly stocks generated or used in this study is available in the Supplementary Table S1.

### Creation of the *PAPLA1* gRNAs transgenic line

*PAPLA1* gRNA target sites were identified using the target prediction tool available at www.shigen.nig.ac.jp/fly/nigfly/cas9. The *PAPLA1*-targeting vector was based on pBFv.U6.2.B^26^. The vector carries two gRNAs, which target *PAPLA1* exon 2 and 7 sequences, separated by 5978 bp (Fig. 1b). This vector was used for transgenesis by custom embryo injection (BestGene Inc.) to generate a stable line co-expressing these two gRNAs. For details on the vector and gRNA line construction see the Supplementary Information.

### CRISPR/Cas9-mediated mutagenesis of the *PAPLA1* gene

The *PAPLA1*^*1*^ and *PAPLA*^*2*^ mutants were generated by CRISPR/Cas9-mediated genomic engineering according to Kondo and Ueda^26^. Fly line expressing the two *PAPLA1* gRNAs was crossed to a line that expressed *nos* > CAS9, to generate F1 founder males, which co-expressed CAS9 and *PAPLA1* gRNAs in the germline. Male progeny of these founders was propagated and screened for the desired large deletions in the *PAPLA1* region. The resulting *PAPLA1*^*1*^ and *PAPLA1*^*2*^ alleles were molecularly characterized and backcrossed into the control genetic background. Details on the mutagenesis, mutation detection and backcrossing are available in the Supplementary Information.

### Generation of the *UAS-PAPLA1* over-expression line

Full-length cDNA representing the *PAPLA1-RA* transcript isoform (clone LD21067) was obtained from BDGP^48^,^49^. The cDNA was excised from the pBluescript_SK(-) backbone and ligated into the pUAST attB vector. The resulting plasmid was used for custom transgenesis by embryo injection (BestGene Inc.), to generate the stock *w*^*1118*^; +/+; *UAS-PAPLA1 /* TM3 *Ser*^*1*^. Details on the construction of the plasmid and overexpression line are available in the Supplementary Information.

### Hatchability measurement

Hatchability was determined as the ratio between the viable and non-viable eggs (i.e. eggs that did not develop into the 1^st^ instar larvae). Eggs were collected from single male-single female crosses of homozygotes; ≥15 parental pairs were used per genotype. Pairs were placed on a standard food supplemented with active yeast, allowed to mate and lay eggs, and flipped to fresh food vials daily. Hatchability was determined daily, approximately 30 h after the end of oviposition. Eggs laid over one week period were analyzed. In total, at minimum 1210 eggs were tested per genotype.

### Viability measurement (larva to adult survival)

Flies were allowed to mate and lay eggs on Petri dishes with standard food supplemented with active yeast. 1^st^ instar larvae were collected from the dishes into vials with standard food, at a density of 50 larvae per 68 ml vial. At least four replicates with 50 larvae each were tested per genotype.

### Developmental rate measurement (time to pupariation)

1^st^ instar larvae were collected as for the viability measurement. At least six replicates of around 50 larvae were tested per genotype. Number of pupariated animals was checked every eight hours, until the last larva entered metamorphosis.

### Determination of pupal volume

Pupal volume was measured using a standard approach^50^; photographs of pupae were taken using stereomicroscope Zeiss Discovery. V8 with Canon EOS-5D Mark II digital camera attached to it and analyzed by ImageJ. Volume was calculated according to the formula 4/3π(L/2)(l/2)^2^ (L = length; l = diameter). At least 16 pupae were measured per genotype.

### Determination of body size based on wing area

Body size was determined based on the wing area^51^ with modifications as described previously^30^. Left wings of 20 males were analyzed per genotype.

### Fecundity assay of *PAPLA1* deficient parents

Homozygous *PAPLA1*^*1*^ and *PAPLA1*^*2*^ virgin females and *w*^*1118*^ control virgin females were collected, and single male-single virgin female intercrosses among the same genotypes were put on standard food supplemented with active yeast. Food was exchanged daily. Daily egg scores were counted over one week. Per genotype, ≥15 couples were tested.

### Testing the male-specific requirements of *PAPLA1* in reproduction

Homozygous *PAPLA1*^*1*^, *PAPLA1*^*2*^ and control virgin males were crossed individually to virgin females of *w*^*1118*^ tester stock. Fecundity and hatchability was determined as described above.

### Testing the female-specific requirements of *PAPLA1* in reproduction

Homozygous *PAPLA1*^*1*^, *PAPLA1*^*2*^ and control virgin females were crossed individually to males of the genetically matched *w*^*1118*^ tester stock (RKF1084). Fecundity and hatchability were determined as described above.

### Imaging of reproductive organs

Male and female reproductive tracts were dissected in PBS, and imaged directly, or after fixation in 4% paraformaldehyde followed by staining with Hoechst 33342 according to a standard protocol. Bright-field images of non-fixed reproductive tracts were taken using stereomicroscope Zeiss Discovery. V8 with Canon EOS-5D Mark II digital camera attached to it. Hoechst 33342-stained samples were imaged using Zeiss Axiophot microscope with Zeiss AxioCam HRC.

### Metabolic rate measurements

Metabolic rate was estimated by respirometry according to Yatsenko *et al*.^52^, with experimental details and modification described previosly^32^. At least five replicates of five flies were tested per genotype.

### ATP measurements

ATP levels were determined by bioluminescence assay using the ATP determination kit (Molecular Probes A22066), as described^53^. Five to six replicates of five flies each were tested per genotype.

### Determination of spontaneous locomotion

Spontaneous locomotion was tested using the *Drosophila* Activity monitoring 2 system (TriKinetics), as described previously^30^. The assay started with 1-day-old flies, and locomotion was monitored over a one-week period. 32 males were tested per each genotype.

### Capillary feeding assay

Food intake measurement was based on the capillary feeding described by Ja *et al*.^54^, with experimental details and modification as described previously^32^. Daily food intake of 20–23 individual males per genotype was monitored over three days, starting one day after eclosion.

### Measurement of lipid, glycogen and protein levels

Lipid, glycogen and protein levels were measured by colorimetric assays from the same starting homogenate as described previously^32^. Four to six replicates of five flies each were tested for each genetic manipulation. Lipid and glycogen content was standardized to the protein levels, and the values were normalized to the values of the genetically matched controls. To ensure direct comparison of body fat storage, *bmm*^*1*^ and *Akh*^*A*^ mutants have been backcrossed into the same genetic background as the *PAPLA1* mutants prior to analysis.

### Testing the changes in the fat reserves upon starvation

One-week old males were collected for colorimetric lipid and protein measurements before starvation (0 h starvation), after starvation on 0.6% agarose medium for 24 h, and after complete starvation to death, respectively. Each replicate consisted of five flies and 4–6 replicates were tested per genotype. The lipid content was standardized to protein levels of the given replicate, and the obtained values were normalized to the 0 h starvation value of the control flies.

### Quantitative real-time PCR

Total RNA was extracted using the Zymo Research Quick-RNA™ MiniPrep kit. Three biological replicates, each consisting of 10 flies were used. cDNA synthesis, qRT-PCR and analyses were done as described in details previously^32^. Information on the primers is available in the Supplement.

### GeneSwitch-mediated genetic manipulations

The GeneSwitch was induced at the age of three days after eclosion, when flies were transferred on standard food supplemented with 200 μM RU-486 (in the figures referred to as RU-486+), or on standard food without RU-486 (in the figures referred as RU-486−). Details on the RU-486 treatment are available in the Supplementary Information.

### Illustration of proteins and their functional domains

Illustrations were done by the IBS Illustrator for Biosequences (http://www.ibs.biocuckoo.org). Protein sequences were obtained from NCBI (https://www.ncbi.nlm.nih.gov/protein), and motifs/domains were found using MOTIF Search (http://www.genome.jp/tools/motif).

### Statistical analyses

Nominal variables were analyzed by the Fischer’s exact test (using GraphPad). Measurement variables were analyzed either by the two-tailed Student’s *t*-test (using Excel) or, when data distribution was not normal, by the Mann-Whitney U test (PAST^55^). Statistical significance of the differences between the samples is indicated by asteriks (*P < 0.05, **P < 0.01, ***P < 0.001). Plotted are means ± SEM.

## Additional Information

**How to cite this article**: Gáliková, M. *et al*. Spastic paraplegia-linked phospholipase *PAPLA1* is necessary for development, reproduction, and energy metabolism in *Drosophila. Sci. Rep.* **7**, 46516; doi: 10.1038/srep46516 (2017).

**Publisher's note:** Springer Nature remains neutral with regard to jurisdictional claims in published maps and institutional affiliations.

## Supplementary Material

Supplementary Information

## References

[b1] Tappia P. S. & Dhalla N. S. Phospholipases in Health and Disease. (Springer Science & Business Media), doi: 10.1007/978-1-4939-0464-8(2014).

[b2] Arrese E. L., Patel R. T. & Soulages J. L. The main triglyceride-lipase from the insect fat body is an active phospholipase A(1): identification and characterization. J. Lipid Res. 47, 2656–2667, doi: 10.1194/jlr.M600161-JLR200 (2006). 17005997

[b3] Richmond G. S. & Smith T. K. Phospholipases A_1_. Int. J. Mol. Sci. 12, 588–612, doi: 10.3390/ijms12010588 (2011). 21340002 PMC3039968

[b4] Tani K., Kogure T. & Inoue H. The intracellular phospholipase A1 protein family. Biomol. Concepts 3, 471–478, doi: 10.1515/bmc-2012-0014 (2012). 25436551

[b5] Mignarri A. . Mitochondrial dysfunction in hereditary spastic paraparesis with mutations in DDHD1/SPG28. J. Neurol. Sci. 362, 287–291, doi: 10.1016/j.jns.2016.02.007 (2016). 26944165

[b6] Schuurs-Hoeijmakers J. H. M. . Mutations in DDHD2, encoding an intracellular phospholipase A(1), cause a recessive form of complex hereditary spastic paraplegia. Am. J. Hum. Genet. 91, 1073–1081, doi: 10.1016/j.ajhg.2012.10.017 (2012). 23176823 PMC3516595

[b7] Fink J. K. Hereditary spastic paraplegia: clinico-pathologic features and emerging molecular mechanisms. Acta Neuropathol. 126, 307–328, doi: 10.1007/s00401-013-1115-8 (2013). 23897027 PMC4045499

[b8] Tesson C., Koht J. & Stevanin G. Delving into the complexity of hereditary spastic paraplegias: how unexpected phenotypes and inheritance modes are revolutionizing their nosology. Hum. Genet. 134, 511–538, doi: 10.1007/s00439-015-1536-7 (2015). 25758904 PMC4424374

[b9] Salinas S., Proukakis C., Crosby A. & Warner T. T. Hereditary spastic paraplegia: clinical features and pathogenetic mechanisms. Lancet Neurol. 7, 1127–1138, doi: 10.1016/S1474-4422(08)70258-8 (2008). 19007737

[b10] Harding A. E. Hereditary ‘pure’ spastic paraplegia: a clinical and genetic study of 22 families. J. Neurol. Neurosurg. Psychiatr. 44, 871–883, doi: 10.1136/jnnp.44.10.871 (1981). 7310405 PMC491171

[b11] McDermott C., White K., Bushby K. & Shaw P. Hereditary spastic paraparesis: a review of new developments. J. Neurol. Neurosurg. Psychiatr. 69, 150–160, doi: 10.1136/jnnp.69.2.150 (2000). 10896685 PMC1737070

[b12] Liguori R. . Impairment of brain and muscle energy metabolism detected by magnetic resonance spectroscopy in hereditary spastic paraparesis type 28 patients with DDHD1 mutations. J. Neurol. 261, 1789–1793, doi: 10.1007/s00415-014-7418-4 (2014). 24989667

[b13] Citterio A. . Mutations in CYP2U1, DDHD2 and GBA2 genes are rare causes of complicated forms of hereditary spastic paraparesis. J. Neurol. 261, 373–381, doi: 10.1007/s00415-013-7206-6 (2014). 24337409

[b14] de Bot S. T. . Rapidly deteriorating course in Dutch hereditary spastic paraplegia type 11 patients. Eur. J. Hum. Genet. 21, 1312–1315, doi: 10.1038/ejhg.2013.27 (2013). 23443022 PMC3798836

[b15] Al-Yahyaee S. . A novel locus for hereditary spastic paraplegia with thin corpus callosum and epilepsy. Neurology 66, 1230–1234, doi: 10.1212/01.wnl.0000208501.52849.dd (2006). 16636240

[b16] Baba T. . Phosphatidic acid (PA)-preferring phospholipase A1 regulates mitochondrial dynamics. J. Biol. Chem. 289, 11497–11511, doi: 10.1074/jbc.M113.531921 (2014). 24599962 PMC4036285

[b17] Arimitsu N. . p125/Sec23-interacting protein (Sec23ip) is required for spermiogenesis. FEBS Lett. 585, 2171–2176, doi: 10.1016/j.febslet.2011.05.050 (2011). 21640725

[b18] Orso G. . Disease-related phenotypes in a *Drosophila* model of hereditary spastic paraplegia are ameliorated by treatment with vinblastine. J. Clin. Invest. 115, 3026–3034, doi: 10.1172/JCI24694 (2005). 16276413 PMC1265857

[b19] Roll-Mecak A. & Vale R. D. The *Drosophila* homologue of the hereditary spastic paraplegia protein, spastin, severs and disassembles microtubules. Curr. Biol. 15, 650–655, doi: 10.1016/j.cub.2005.02.029 (2005). 15823537

[b20] Baxter S. L., Allard D. E., Crowl C. & Sherwood N. T. Cold temperature improves mobility and survival in *Drosophila* models of autosomal-dominant hereditary spastic paraplegia (AD-HSP). Dis. Model Mech. 7, 1005–1012, doi: 10.1242/dmm.013987 (2014). 24906373 PMC4107329

[b21] Füger P. . Spastic paraplegia mutation N256S in the neuronal microtubule motor KIF5A disrupts axonal transport in a *Drosophila* HSP model. PLoS Genet. 8, e1003066, doi: 10.1371/journal.pgen.1003066 (2012). 23209432 PMC3510046

[b22] Liebl F. L. W. . Genome-wide P-element screen for *Drosophila* synaptogenesis mutants. J. Neurobiol. 66, 332–347, doi: 10.1002/neu.20229 (2006). 16408305 PMC1626350

[b23] Kunduri G. . Phosphatidic acid phospholipase A1 mediates ER-Golgi transit of a family of G protein-coupled receptors. J. Cell Biol. 206, 79–95, doi: 10.1083/jcb.201405020 (2014). 25002678 PMC4085702

[b24] Hanlon C. D. & Andrew D. J. Outside-in signaling–a brief review of GPCR signaling with a focus on the *Drosophila* GPCR family. J. Cell. Sci. 128, 3533–3542, doi: 10.1242/jcs.175158 (2015). 26345366 PMC4610211

[b25] Nässel D. R. & Winther A. M. E. *Drosophila* neuropeptides in regulation of physiology and behavior. Prog. Neurobiol. 92, 42–104, doi: 10.1016/j.pneurobio.2010.04.010 (2010). 20447440

[b26] Kondo S. & Ueda R. Highly Improved Gene Targeting by Germline-Specific Cas9 Expression in *Drosophila*. Genetics 195, 715–721, doi: 10.1534/genetics.113.156737 (2013). 24002648 PMC3813859

[b27] Ravi Ram K. & Wolfner M. F. Seminal influences: *Drosophila* Acps and the molecular interplay between males and females during reproduction. Integr. Comp. Biol. 47, 427–445, doi: 10.1093/icb/icm046 (2007). 21672851

[b28] McCall K. Eggs over easy: cell death in the *Drosophila* ovary. Dev. Biol. 274, 3–14, doi: 10.1016/j.ydbio.2004.07.017 (2004). 15355784

[b29] Grönke S. . Brummer lipase is an evolutionary conserved fat storage regulator in *Drosophila*. Cell Metab. 1, 323–330, doi: 10.1016/j.cmet.2005.04.003 (2005). 16054079

[b30] Gáliková M. . Energy Homeostasis Control in *Drosophila* Adipokinetic Hormone Mutants. Genetics 201, 665–683, doi: 10.1534/genetics.115.178897 (2015). 26275422 PMC4596676

[b31] Tricoire H. . The steroid hormone receptor EcR finely modulates *Drosophila* lifespan during adulthood in a sex-specific manner. Mech. Ageing Dev. 130, 547–552, doi: 10.1016/j.mad.2009.05.004 (2009). 19486910

[b32] Gáliková M., Klepsatel P., Xu Y. & Kühnlein R. P. The obesity‐related Adipokinetic hormone controls feeding and expression of neuropeptide regulators of *Drosophila* metabolism. Eur. J. Lipid. Sci. Tech., doi: 10.1002/ejlt.201600138 (2016).

[b33] Suh J. M. . Adipose is a conserved dosage-sensitive antiobesity gene. Cell Metab. 6, 195–207, doi: 10.1016/j.cmet.2007.08.001 (2007). 17767906 PMC2587167

[b34] Broughton S. J. . Longer lifespan, altered metabolism, and stress resistance in *Drosophila* from ablation of cells making insulin-like ligands. Proc. Natl. Acad. Sci. USA 102, 3105–3110, doi: 10.1073/pnas.0405775102 (2005). 15708981 PMC549445

[b35] Grönke S., Clarke D.-F., Broughton S., Andrews T. D. & Partridge L. Molecular evolution and functional characterization of *Drosophila* insulin-like peptides. PLoS Genet. 6(2), e100085, doi: 10.1371/journal.pgen.1000857 (2010). PMC2829060 20195512

[b36] Buch S., Melcher C., Bauer M., Katzenberger J. & Pankratz M. J. Opposing effects of dietary protein and sugar regulate a transcriptional target of *Drosophila* insulin-like peptide signaling. Cell Metab. 7, 321–332, doi: 10.1016/j.cmet.2008.02.012 (2008). 18396138

[b37] Synofzik M. . PNPLA6 mutations cause Boucher-Neuhauser and Gordon Holmes syndromes as part of a broad neurodegenerative spectrum. Brain 137, 69–77, doi: 10.1093/brain/awt326 (2014). 24355708 PMC3891450

[b38] Yadav P. K. & Rajasekharan R. Misregulation of a DDHD Domain-containing Lipase Causes Mitochondrial Dysfunction in Yeast. J. Biol. Chem. 291, 18562–18581, doi: 10.1074/jbc.M116.733378 (2016). 27402848 PMC5000100

[b39] Tesson C. . Alteration of fatty-acid-metabolizing enzymes affects mitochondrial form and function in hereditary spastic paraplegia. Am. J. Hum. Genet. 91, 1051–1064, doi: 10.1016/j.ajhg.2012.11.001 (2012). 23176821 PMC3516610

[b40] Inloes J. M. . The hereditary spastic paraplegia-related enzyme DDHD2 is a principal brain triglyceride lipase. Proc. Natl. Acad. Sci. USA 111, 14924–14929, doi: 10.1073/pnas.1413706111 (2014). 25267624 PMC4205627

[b41] Baumbach J. . A *Drosophila in vivo* screen identifies store-operated calcium entry as a key regulator of adiposity. Cell Metab. 19, 331–343, doi: 10.1016/j.cmet.2013.12.004 (2014). 24506874

[b42] Pospisilik J. A. . *Drosophila* genome-wide obesity screen reveals hedgehog as a determinant of brown versus white adipose cell fate. Cell 140, 148–160, doi: 10.1016/j.cell.2009.12.027 (2010). 20074523

[b43] Rossi A. . Genetic compensation induced by deleterious mutations but not gene knockdowns. Nature 524, 230–233, doi: 10.1038/nature14580 (2015). 26168398

[b44] Di Cara F. & King-Jones K. The Circadian Clock Is a Key Driver of Steroid Hormone Production in *Drosophila*. Curr. Biol. 26, 2469–2477, doi: 10.1016/j.cub.2016.07.004 (2016). 27546572

[b45] Danielsen E. T. & Rewitz K. F. Developmental Biology: When Less Damage Causes More Harm. Curr. Biol. 26, R855–858, doi: 10.1016/j.cub.2016.07.068 (2016). 27676307

[b46] Gonzalez M. . Mutations in phospholipase DDHD2 cause autosomal recessive hereditary spastic paraplegia (SPG54). Eur. J. Hum. Genet. 21, 1214–1218, doi: 10.1038/ejhg.2013.29 (2013). 23486545 PMC3798837

[b47] Kumar K. R. . Defining the genetic basis of early onset hereditary spastic paraplegia using whole genome sequencing. Neurogenetics 17, 265–270, doi: 10.1007/s10048-016-0495-z (2016). 27679996 PMC5061846

[b48] Stapleton M. . The *Drosophila* gene collection: identification of putative full-length cDNAs for 70% of D. melanogaster genes. Genome Res. 12, 1294–1300, doi: 10.1101/gr.269102 (2002). 12176937 PMC186637

[b49] Lin M. F. . Revisiting the protein-coding gene catalog of *Drosophila* melanogaster using 12 fly genomes. Genome Res. 17, 1823–1836, doi: 10.1101/gr.6679507 (2007). 17989253 PMC2099591

[b50] Delanoue R., Slaidina M. & Léopold P. The steroid hormone ecdysone controls systemic growth by repressing dMyc function in *Drosophila* fat cells. Dev. Cell 18, 1012–1021, doi: 10.1016/j.devcel.2010.05.007 (2010). 20627082

[b51] Klepsatel P., Gáliková M., Huber C. D. & Flatt T. Similarities and differences in altitudinal versus latitudinal variation for morphological traits in *Drosophila melanogaster*. Evolution 68, 1385–1398, doi: 10.1111/evo.12351 (2014). 24410363

[b52] Yatsenko A. S., Marrone A. K., Kucherenko M. M. & Shcherbata H. R. Measurement of metabolic rate in *Drosophila* using respirometry. J. Vis. Exp. e51681, doi: 10.3791/51681 (2014). 24998593 PMC4205100

[b53] Tennessen J. M., Barry W. E., Cox J. & Thummel C. S. Methods for studying metabolism in *Drosophila*. Methods 68, 105–115, doi: 10.1016/j.ymeth.2014.02.034 (2014). 24631891 PMC4048761

[b54] Ja W. W. . Prandiology of *Drosophila* and the CAFE assay. Proc. Natl. Acad. Sci. USA 104, 8253–8256, doi: 10.1073/pnas.0702726104 (2007). 17494737 PMC1899109

[b55] Hammer Ø., Harper D. A. T. & Ryan P. D. Paleontological Statistics Software: Package for Education and Data Analysis. Palaeontol. Electron. 4, 9 (2001).

