# Supplementary material for: Spastic paraplegia-linked phospholipase PAPLA1 is necessary for development, reproduction, and energy metabolism in Drosophila
Source: Sci Rep. 2017 Apr 19;7:46516. doi: 10.1038/srep46516 (PMC5395975; doi:10.1038/srep46516)
Supplement: Supplementary Information [file srep46516-s1.pdf]

Supplementary Information

Spastic paraplegia-linked phospholipase *PAPLA1* is necessary for development, reproduction, and energy metabolism in *Drosophila*

Martina Gáliková, Peter Klepsatel, Judith Münch, and Ronald P. Kühnlein

Supplementary Material & Methods

Fly food

Standard *Drosophila* food of the following composition was used: 5.43 g agar, 15.65 g yeast, 8.7 g soy flour; 69.57 g maize flour, 19.13 g beet syrup, 69.57 g malt, 5.43 ml propionic acid and 1.3 g methyl 4-hydroxybenzoate per 1L of medium.

Fly husbandry

Larval and adult density was controlled as follows: approximately 150 eggs were seeded per one 68 ml vial, and adult flies were collected within 24 h after eclosion. If not described otherwise, approximately 50 females + 50 males were housed per one 68 ml vial. Adult flies were flipped on fresh media every 2nd day. For the analyses of the mutants, one-week-old males were used, if not specified otherwise. For the analyses employing the GeneSwitch system, male flies were used that had been reared on the RU-486 food for six days.

Creation of the *PAPLA1* gRNAs transgenic line

The gRNA1 oligonucleotides RKO897+898 (targeting 5' region of *PAPLA1*; see Supplementary Table S3) were annealed and cloned into *BbsI*–digested pBFv.U6.2, giving rise to pBFv.U6.2_*PAPLA1*.gRNA1 vector. Similarly, gRNA2 oligonucleotides RKO903+904 (targeting 3' region of *PAPLA1;* see Supplementary Table S3) were annealed and cloned into the *BbsI*–digested pBFv.U6.2B vector resulting in pBFv.U6.2B_*PAPLA1*.gRNA2 vector. Subsequently, gRNA1 was excised from pBFv.U6.2_*PAPLA1*.gRNA1 via *EcoRI* and *NotI* and sub-cloned into the pBFv.U6.2B_*PAPLA1*.gRNA2. The resulting plasmid pBFv.U6.2B_*PAPLA1*.gRNA1+2 (MG458) was injected by BestGene Inc. into the fly line *M{3xP3-RFP.attP}ZH-86Fb* (BDSC24749), landing site 86F8. The F1 generation was crossed to y^1, sc^* v^1; Dr^1, e^1/TM3, Sb^1 (BDSC32261) and selected for the presence of v^+, to establish the following transgenic line with ubiquitous expression of *PAPLA1* gRNA1+2 (done by BestGene): y^1 sc^* v^1; *P{*v^+*; BFv-U6.2B_PAPLA1.gRNA1+2}att*P.

<u>Generation of the *PAPLA1* mutant lines</u>

For the induction of *PAPLA1* mutations, *y*^{1} *sc* v*^{1}*; P{v*$^{+}$*; BFv-U6.2B_PAPLA1.gRNA1+2}att*P was crossed to *P{ry*$^{+t7.2}$*=hsFLP}1, y*^{1} *w*^{1118}*; P{y*$^{+t7.7}$ *w*$^{+mC}$*=UAS*-Cas9.P*}attP2, P{w*$^{+mC}$=GAL4::VP16-*nos.UTR}CG6325*^{MVD1} to generate founder males that co-expressed CAS9 and the *PAPLA1* gRNAs in the germline. Founder males were crossed to *w*; Kr*^{If1} / CyO*; D*^{1} / TM3*, Ser*^{1} females. Male *PAPLA1* candidate mutants (genotype: *w*; PAPLA1**$^{?}$ / CyO*; P{v*; BFv-U6.2B_PAPLA1.gRNA1+2}att*P / TM3*, Ser*^{1}) were mated to *w*; Sp*^{1} / CyO*, P{w*$^{+mC}$*=Dfd-EYFP}2* females in single male crosses, and after mating, candidate mutant males were individually genotyped for deletions in the *PAPLA1* region by PCR genotyping, using primers RKO916 and RKO917 (see Supplementary Table S3) that flank the gRNA1 and gRNA2 target sites, respectively. PCR genotyping revealed large deletions in four of the tested males. Progeny of these males was used to establish stable lines, sequencing of which confirmed four deletion alleles of *PAPLA1* gene. Two of these deletions, *PAPLA1*^{1} and *PAPLA1*^{2}, were further used in this study. Since life history traits and many physiological parameters are prone to confounding genetic background effects, the *PAPLA1*^{1} and *PAPLA1*^{2} alleles were backcrossed into the standard *w*^{1118} genetic background (VDRC60000; RKF1084) for nine generations. During the backcrossing, the mutations were tracked by PCR genotyping using primers RKO916 and RKO917. Simultaneous backcrossing of the *PAPLA1* alleles, the *Akh*^{A1} and the *bmm*^{1}[2] mutations allowed direct comparisons of loss-of-functions effects of all of these genes in the identical genetic background.

<u>Generation of the *UAS-PAPLA1* over-expression line</u>

Full-length cDNA of *PAPLA1-RA* (6588 bp, BDGP clone LD21067) was excised from the pBluescript_SK(-) backbone by *SmaI* and *XhoI* and ligated into the pUAST attB vector[3] treated as follows: first, the vector was linearized by *EcoRI,* ends were blunted by Klenow fragment, and cleaved by *XhoI*. Sequence of the cloned *PAPLA1* over-expression construct within this plasmid was confirmed by sequencing (Eurofins Genomic). The resulting plasmid pUAST attB_*PAPLA1* (MG465) was injected by BestGene Inc. into the *attP* strain *M{3xP3-RFP.attP}ZH-86Fb* (BDSC24749), and the stable line *w*^{1118}*; +/+; UAS-PAPLA1 /* TM3 *Ser*^{1} floating (MGF1601) was established.

<u>GeneSwitch-mediated genetic manipulations</u>

To avoid the confounding effects of genetic background on fly reproduction or metabolism, we used either mutants backcrossed to a common genetic background (see above), or the GeneSwitch method. This system is based on the induction of the UAS-GAL4 system by feeding flies with the drug mifepristone (RU-486)[4,5]. Thus, the experimental and control animals are genetically identical, and differ only by the presence or absence of the RU-486

drug in the food[4,5]. RU-486 itself does not significantly affect fly physiology, and the RU-486 based Gene Switch is thus a commonly used tool to study various traits[4,5], including lipid and glycogen storage,[6-10]. Next to others reports, our previous work[10] also confirmed that RU-486 itself does not affect energy storage. The experiment was conducted as described previously[9,10]; GeneSwitch was induced at the age of three days after eclosion, when flies were transferred to standard food supplemented with 200 µM RU-486 (dilution from 20 mM stock solution in 96% ethanol; in the figures referred as RU-486 +), or to the standard food with the 0 µM RU-486 (vehicle, i.e. the 96% ethanol; in the figures referred as RU-486 -). Food was exchanged every second day, flies were used for experiments after six days of feeding with or without RU-486.

Table S1: List of fly lines used in this study

**Genotype**	**Internal stock #**	**External stock # / reference**
*y^1 sc*y1 y^1 sc* v^1; v^1; P{v^+; BFv-6.2B_PAPLA1.gRNA1+2}attP*	MGF1497	This study
w; Kr^{If-1}/ CyO; D^1 / TM3, Ser^1*	RKF1365	BDSC7198
P{$ry^{+t7.2}$=hsFLP}1, y^1 w^{1118}; P{$y^{+t7.7}$ w^{+mC}=UAS-Cas9.P}attP2, P{w^{+mC}=GAL4::VP16-nos.UTR}$CG6325^{MVD1}$	MGF1476	BDSC54593,[11]
w; Sp^1 / CyO, P{w[+mC]=Dfd-EYFP}2*	-	Gift from Dr. Ralf Pflanz
w^{1118}; PAPLA1^1/ CyO; P{w[+mC]=Dfd-EYFP}2	MGF1642	This study
w^{1118}; PAPLA1^2/ CyO; P{w[+mC]=Dfd-EYFP}2	MGF1643	This study
w^{1118}; Akh^A	MGF1629	[1]
w^{1118}; bmm^1 / TM3, Ser^1	RKF1566	This study; derived from *bmm^1* [2] and *TM3, Ser^1* backcrossed to RKF1084
w^{1118}	MGF1638	This study; genetically matched control, derived from backcrossing to RKF1084
w^{1118}; +/+; UAS-PAPLA1 / TM3 Ser^1 (floating balancer)	MGF1601	This study
w^{1118}	RKF1084	VDRC60000 (line used for backcrossing), *PAPLA1$^+$* tester stock
w; P{Switch1}FBI-26; UAS-GFP*	RKF1045	[6]

w^{1118}*; daughterless-GeneSwitch*	MGF1663	[13]
w; +/+; P{Lpp-GAL4.B}/TM3, Sb* float*	RKF1421	[14]
w^{1118}*; P{KK100753}VIE-260B (PAPLA1* RNAi line1)	MGF1503	VDRC108121
w^{1118}*; P{GD13962}v35957 260B (PAPLA1* RNAi line2)	MGF1504	VDRC35957
w^{1118}*;UAS-AkhR RNAi / TM3,* Ser^{1}	MGF1635	[9]
w^{1118}*; P{GD5139}v37880 (bmm* RNAi)	JBF1454	VDRC37880
w; + / +; P{*w^{+mC}*bmm[Scer\UAS]=UAS-bmm}#2d*	SGF533	[2]
w^{1118}*; P{*w^{+mC}*=UAS-Akh.L}2*	MGF1633	[9]

Table S2: List of used qPCR primers

Gene	Sequence / reference number	Internal nr.	Reference/source
bmm	QuantiTect Primer#QT00964460	YXO1169	Qiagen
AkhR	QuantiTect Primer#QT00931210	RWO719	Qiagen
mdy	QuantiTect Primer#QT00501774	RKO725	Qiagen
Desat1	F: TGAACAGTGCTGCCCACAAG<br>R: GGAGATGTTCTCCGAAGGATTG	RKO740<br>RKO741	[15]
ACC	**F:** GTGCAACTGTTGGCAGATCAGT<br>R: TTTCTGATGACGACGCTGGAT	RKO738<br>RKO739	[15]
FASN1	***F:*** CTCCACCATCGAGGAGTTCA<br>R: CTTGAGCTTGCCAATCCTGT	YXO1187<br>YXO1188	[16]
Ilp2	F: ACGAGGTGCTGAGTATGGTGTGCG<br>R: CACTTCGCAGCGGTTCCGATATCG	JBO875<br>JBO876	[17]
Ilp3	QuantiTect Primer#QT00961737	RKO968	Qiagen
Ilp5	F: GAGGCACCTTGGGCCTATTC<br>R: CATGTGGTGAGATTCGGAGCTA	JBO886<br>JBO887	[18]
tobi	QuantiTect Primer#QT00982646	RKO969	Qiagen
Pgm	QuantiTect Primer#QT00512568	AAO521	Qiagen
PAPLA1	QuantiTect Primer#QT00932197	MGO1027	Qiagen
Act5C	F: GTGCACCGCAAGTGCTTCTAA<br>R: TGCTGCACTCCAAACTTCCAC	RKO744<br>RKO745	[19]
RpL32	QuantiTect Primer#QT00985677	RKO977	Qiagen
Thor	F: CATGCAGCAACTGCCAAATC<br>R: CCGAGAGAACAAACAAGGTGG	JBO753<br>JBO754	[20]
l(2)efl	F: AGGACGATGTGACCGTGTC<br>R: CGAAGCAGACGCGTTTATCC	MGO1143<br>MGO1144	[21]

Table S3: List of additional oligonucleotides and primers used for cloning and genotyping

Gene/ construct	Sequence	Internal nr.	Note
PAPLA1 **gRNA1 For.**	CTTCGAGGATTCCGTTGGCGCAGG	RKO897	Annealing with RKO898 and subsequent cloning into gRNA vector
PAPLA1 **gRNA1 Rev.**	AAACCCTGCGCCAACGGAATCCTC	RKO898	Annealing with RKO897 and subsequent cloning into gRNA vector
PAPLA1 **gRNA2 For.**	CTTCGGGTCGCTCGATGGTCATTG	RKO903	Annealing RKO904 and subsequent cloning into gRNA vector
PAPLA1 **gRNA2 Rev.**	AAACCAATGACCATCGAGCGACCC	RKO904	Annealing RKO903 and subsequent cloning into gRNA vector
genomic PAPLA1	F: GTCCAGTTCCACCTTCGATTC<br>R: GGTCTCATGGGTGCCAATCC	RKO916<br>RKO917	Identification and backcrossing of the deletion alleles

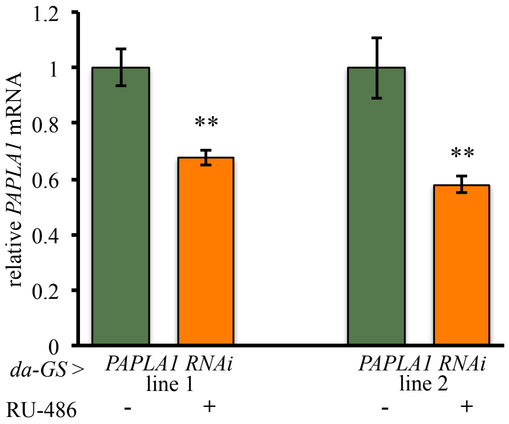

Figure S1 Ubiquitous induction of *PAPLA1* RNAi lines efficiently decrease *PAPLA1* mRNA levels. Two-tailed Students *t*–test: $P < 0.01$.

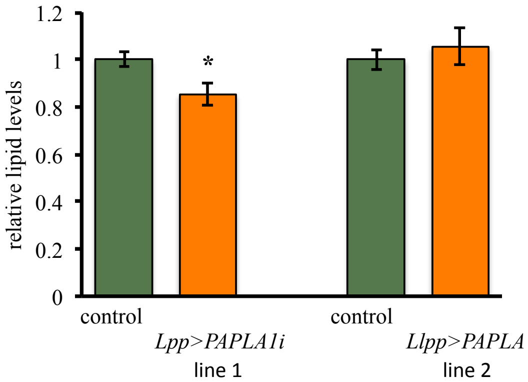

Figure S2 Little effect on body fat content in response to fat body-specific RNAi against *PAPLA1* gene knockdown throughout ontogenesis (*Lpp*-GAL4). Small body fat reduction using RNAi line 1. Two-tailed Students *t*–test: $P < 0.05$. No effect on body fat using line 2. Two-tailed Student`s *t*–test: $P > 0.05$.

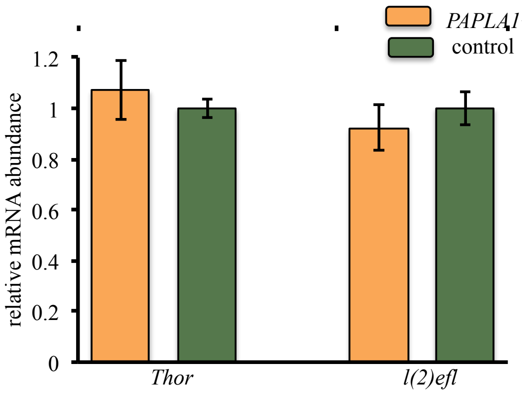

Figure S3 The expression of insulin-like signaling targets *Thor* and *l(2)efl* is not changed in *PAPLA1* deficient mutants compared to controls. Two-tailed Students *t*–test: $P >$ 0.05.

Supplementary references

1. Gáliková, M. *et al.* Energy Homeostasis Control in Drosophila Adipokinetic Hormone Mutants. *Genetics* **201,** 665-683, doi: 10.1534/genetics.115.178897 (2015).
2. Grönke, S. *et al.* Brummer lipase is an evolutionary conserved fat storage regulator in Drosophila. *Cell Metab.* **1,** 323-330, doi: 10.1016/j.cmet.2005.04.003 (2005).
3. Bischof, J., Maeda, R. K., Hediger, M., Karch, F. & Basler, K. An optimized transgenesis system for Drosophila using germ-line-specific phiC31 integrases. *Proc. Natl. Acad. Sci. USA* **104,** 3312–3317, doi : 10.1073/pnas.0611511104 (2007).
4. Roman, G., Endo, K., Zong, L. & Davis, R. L. P[Switch], a system for spatial and temporal control of gene expression in Drosophila melanogaster. *Proc. Natl. Acad. Sci. USA* **98,** 12602–12607, doi: 10.1073/pnas.221303998 (2001).
5. Osterwalder, T., Yoon, K.S., White, B.H. & Keshishian, H. A conditional tissue-specific transgene expression system using inducible GAL4. *Proc. Natl. Acad. Sci. USA* **98,** 12596-601, doi: 10.1073/pnas.221303298 (2001).
6 Suh, J. M. *et al.* Adipose is a conserved dosage-sensitive antiobesity gene. *Cell Metab.* **6,** 195-207, doi: 10.1016/j.cmet.2007.08.001 (2007).
7. Xu, K., Zheng, X. & Sehgal, A. Regulation of feeding and metabolism by neuronal and peripheral clocks in Drosophila. *Cell Metab.* **8**, 289-300, doi:10.1016/j.cmet.2008.09.006 (2008).
8. Bai, H., Kang, P. & Tatar, M. Drosophila insulin-like peptide-6 (dilp6) expression from fat body extends lifespan and represses secretion of Drosophila insulin-like peptide-2 from the brain. *Aging Cell* **11**, 978-985, doi: 10.1111/acel.12000 (2012).
9. Gáliková, M., Klepsatel, P., Xu, Y. & Kühnlein, R. P. The obesity−related Adipokinetic hormone controls feeding and expression of neuropeptide regulators of Drosophila metabolism. *Eur. J. Lipid. Sci. Tech*, doi:10.1002/ejlt.201600138 (2016).
10. Klepsatel P., Gáliková M**.**, Xu Y. & Kühnlein R.P. Thermal stress depletes energy reserves in *Drosophila*. *Sci Rep.* Sep 19;6:33667. doi: 10.1038/srep33667.
11. Port, F., Chen, H. M., Lee, T. & Bullock, S. L. Optimized CRISPR/Cas tools for efficient germline and somatic genome engineering in Drosophila. *Proc. Natl. Acad. Sci. USA* **111,** E2967–E2976, doi: 10.1073/pnas.1405500111 (2014).
12. Suh, J. M. *et al.* Adipose is a conserved dosage-sensitive antiobesity gene. *Cell Metab.* **6,** 195-207, doi: 10.1016/j.cmet.2007.08.001 (2007).
13. Tricoire, H. *et al.* The steroid hormone receptor EcR finely modulates Drosophila lifespan during adulthood in a sex-specific manner. *Mech. Ageing Dev.* **130,** 547–552, doi: 10.1016/j.mad.2009.05.004 (2009).

14 Brankatschk, M. & Eaton, S. Lipoprotein particles cross the blood-brain barrier in Drosophila. *J. Neurosci.* **30,** 10441–10447, doi: 10.1523/JNEUROSCI.5943-09.2010 (2010).

15. Seegmiller, A. C. *et al.* The SREBP Pathway in Drosophila. *Dev. Cell* **2,** 229–238, doi: 10.1016/S1534-5807(01)00119-8 (2002).

16. Mattila, J. *et al.* Mondo-Mlx Mediates Organismal Sugar Sensing through the Gli-Similar Transcription Factor Sugarbabe. *Cell Rep.* **13,** 350–364, doi: 10.1016/j.celrep.2015.08.081 (2015).

17. Baumbach, J. *et al.* A Drosophila in vivo screen identifies store-operated calcium entry as a key regulator of adiposity. *Cell Metab.* **19,** 331–343, doi: 10.1016/j.cmet.2013.12.004 (2014).

18. Broughton, S. J. *et al.* Longer lifespan, altered metabolism, and stress resistance in Drosophila from ablation of cells making insulin-like ligands. *Proc. Natl. Acad. Sci. USA* **102,** 3105–3110, doi: 10.1073/pnas.0405775102 (2005).

19. Bauer, R., Voelzmann, A. & Breiden, B. Schlank, a member of the ceramide synthase family controls growth and body fat in Drosophila. *EMBO J.* **28**, 3706-3716, doi: 10.1038/emboj.2009.305 (2009).

20. Fuss, B., Becker, T., Zinke, I. & Hoch, M. The cytohesin Steppke is essential for insulin signalling in Drosophila. *Nature* **444,** 945–948, doi: 10.1038/nature05412 (2006).

21. Flatt, T. *et al.* Drosophila germ-line modulation of insulin signaling and lifespan. *Proc. Natl. Acad. Sci. USA* **105,** 6368–6373, doi: 10.1073/pnas.0709128105 (2008).
